# Two promising candidates for paratransgenesis, *Elizabethkingia* and *Asaia*, increase in both sexes of *Anopheles gambiae* mosquitoes after feeding

**DOI:** 10.1186/s12936-024-04870-w

**Published:** 2024-02-12

**Authors:** Richardson K. Egyirifa, Jewelna Akorli

**Affiliations:** Department of Parasitology, Noguchi Memorial Institute for Medical Research, University of Ghana, P.O. Box LG 581, Legon, Accra, Ghana

**Keywords:** Mosquito microbiota, *Anopheles gambiae*, *Asaia siamensis*, *Elizabethkingia meningoseptica*, Bio-control

## Abstract

**Background:**

The male mosquito microbiome may be important for identifying ideal candidates for disease control. Among other criteria, mosquito-associated symbionts that have high localization in both male and female mosquitoes and are transmissible through both vertical and sexual routes are desirable. However, mosquito microbiome studies have mainly been female-focused. In this study, the microbiota of male and female *Anopheles gambiae* sensu lato (s.l.) were compared to identify shared or unique bacteria.

**Methods:**

Late larval instars of *Anopheles* mosquitoes were collected from the field and raised to adults. Equal numbers of males and females of 1-day-old non-sugar-fed, 4–5-day-old sugar-fed and post-blood-fed females were randomly selected for whole-body analyses of bacteria 16S rRNA.

**Results:**

Results revealed that male and female mosquitoes generally share similar microbiota except when females were blood-fed. Compared to newly emerged unfed mosquitoes, feeding on sugar and/or blood increased variability in microbial composition (⍺-diversity), with a higher disparity among females (39% *P* = 0.01) than in males (29% *P* = 0.03). *Elizabethkingia meningoseptica* and *Asaia siamensis* were common discriminants between feeding statuses in both males and females. While *E. meningoseptica* was particularly associated with sugar-fed mosquitoes of both sexes and sustained after blood feeding in females, *A. siamensis* was also increased in sugar-fed mosquitoes but decreased significantly in blood-fed females (LDA score > 4.0, *P* < 0.05). Among males, *A. siamensis* did not differ significantly after sugar meals.

**Conclusions:**

Results indicate the opportunities for stable infection in mosquitoes should these species be used in bacteria-mediated disease control. Further studies are recommended to investigate possible host-specific tissue tropism of bacteria species which will inform selection of the most appropriate microbes for effective transmission-blocking strategies.

**Supplementary Information:**

The online version contains supplementary material available at 10.1186/s12936-024-04870-w.

## Background

Malaria control has seen significant success with the use of chemical-based interventions such as insecticide spraying, insecticide-treated bed nets and larviciding, and these remain the major strategies for vector control [1]. New vector control tools are however being considered [2] as widespread increase in insecticide resistance challenges the continuous use of the existing ones to achieve further decline in disease prevalence. In populations where malaria is endemic, the increased burden placed on fragile health systems by emerging epidemics and pandemics further disrupts the sustainability of malaria control programmes, resulting in a rebound in disease cases [3]. In 2022, the World Health Organization (WHO) reported an increase in malaria cases by 2 million during the period of the COVID-19 pandemic, with the heaviest burden still attributed to sub-Saharan Africa [4]. Future pandemics could lead to massive interruptions in malaria control activities thus, supporting the urgent request for tools that are self-propagating, such as gene drives and bacteria-mediated mechanisms.

Over 400 *Anopheles* mosquitoes are known, yet 30–40 are competent vectors of malaria. In sub-Saharan Africa, members of the *Anopheles gambiae* and *Anopheles funestus* complexes are the most competent vectors and, many of the current malaria vector control efforts are based on their vector biology, ecology and behaviour [5]. For example, treated bed nets prevent endophagic night-biting mosquitoes, such as *Anopheles gambiae* and *Anopheles coluzzii* access to the human host while sleeping, and indoor residual spraying kill indoor-resting (endophilic) species on contact or repel them from entering human habitations. However, evidence points to significant shifts in biting and resting behaviours in these *Anopheles* species [6–8]. Subsequently, there is an increase in reported outdoor biting rates [9–11], calling for new tools that would control outdoor-acquired transmission [6–8].

Several new control interventions are at various stages of development and evaluation [12]. Among these is the proposed use of microbes to control human parasites within the mosquito vector. A few bacteria species isolated from mosquitoes have been reported to antagonize the mosquito stages of *Plasmodium* while presenting little or no fitness cost to the insect host [13–17]. Microbiomes have been shown to be less varied between mosquito species in natural populations than among individuals of the same species [18], suggesting selecting microbes that are common to most species will be useful transmission-blocking bioagents in most, if not all, of the major *Anopheles* vectors. However, this needs to be comprehensively investigated.

*Serratia*, *Enterobacter*, *Asaia*, *Elizabethkingia* and *Pseudomonas* are a few of the bacterial species that are commonly identified as part of the *Anopheles* microbiome [19] and are potential candidates for bacteria-mediated disease control. They are particularly predominant in the midgut and reproductive tissues, making them applicable through feeding strategies such as sugar baits [20, 21]. The large-scale field implementation of microbes for the control of adult mosquitoes is yet to be realized for several reasons including identifying the most cost-effective and sustainable symbiont to use, efficient mode of delivery, and environmental and ecological considerations [22, 23].

Until recently many studies have focused on identifying symbionts in female mosquitoes, justified by the fact that these seek blood meals and transmit diseases. Therefore bacteria-mediated methods geared towards parasite transmission blocking will be most effective if candidate microbes are obtained from female mosquitoes. However, by virtue of their common position within the food chain and the possibility of sexual transfer of microbes [24–27], males are also important in identifying the most appropriate candidate bacteria for alternative disease control mechanisms. In addition, males can also be good dissemination methods for introducing the selected bacteria into the mosquito population as it is done in *Aedes*-*Wolbachia* campaigns [28, 29]. In this study, the dynamics of the bacterial symbionts in both male and female adult *Anopheles gambiae* were investigated considering their feeding histories, with the aim of assessing the most sustainable microbial candidate. The results were discussed with reference to implications towards the implementation of microbial-mediated strategies.

## Methods

### Mosquito samples

Late (3rd and 4th) instars and pupae of *Anopheles* mosquitoes were collected from a breeding site in Accra, Ghana and returned to the insectary in portions of the field water in plastic containers. The larvae were fed with fish meal (TetraMin Tropical Flakes) ad libitum, and pupae were picked and placed into cages to emerge. 40 each of 1-day-old non-sugar-fed male and female adults were aspirated into tubes containing 70% ethanol and stored. The remaining adult mosquitoes were fed with 10% sugar solution through cotton balls for a period of 4–5 days and, 35 males and 40 females were picked and stored as previously described. The remaining female adult mosquitoes were given an animal blood meal and non-blood engorged individuals were taken out of the cage. 40 blood-fed females were transferred into tubes and stored.

### DNA and sequencing

Prior to DNA extraction, the mosquitoes were surface sterilized in 5% bleach, then 70% ethanol and finally washed in 1× sterile PBS. DNA was extracted from whole mosquitoes in pools of 5 according to sex and feeding status (unfed, sugar-fed and blood-fed) using a Qiagen DNA Blood and Tissue kit (Hilden, Germany). A negative control (extraction reagents without a mosquito sample) was included during the DNA extraction process to check for contamination in downstream analyses. DNA samples were submitted to a sequencing facility for paired-end 16S amplicon sequencing of bacterial V3–V4 variable region on an Illumina platform.

### Sequence analyses

A total of 7,188,650 paired-end, demultiplexed and pre-cleaned reads were obtained for 40 samples (including the negative control) from the sequencing facility (Additional file 1). Sequence analyses were performed using Quantitative Insights Into Microbial Ecology (QIIME)-2 software version 2023.7 [30, 31]. The sequences were trimmed according to base quality scores, denoised to remove single-end reads, join sequence pairs, and remove chimaeras using *dada2* [32] and *vsearch* [33]. The resulting sequences were clustered at 97% similarity using the *vsearch* algorithm [33] and taxonomically identified against the SILVA 138.1 SSU rRNA database [34–36]. Sequences that were identified as Eukaryota, Archaea and Chloroplasts were filtered out and a rooted phylogenetic tree was constructed. A *phyloseq* object was constructed in R statistical software [37] using the filtered operational taxonomic unit (OTU) table and rooted tree. Potential sequence contaminants were evaluated and removed using *decontam* package [38], considering as contaminants all bacterial taxa that were more prevalent in the negative control than in test samples. Following the identification and removal of contaminants, the negative control was excluded from subsequent analyses to obtain a ‘clean’ *phyloseq* object.

To remove singleton taxa, the ‘clean’ *phyloseq* object was filtered to remove taxa not observed more than once in at least 5% of the samples. OTUs were rarefied for alpha and beta diversity analyses. Taxa richness (observed number of bacteria taxa), evenness and an estimate of within-group variability that considers both richness and evenness (Shannon–Wiener index) were compared between males and females, and their feeding status. A pairwise Wilcox test was employed to estimate the significance of observed differences between groups. Compositional and taxa phylogenetic differences between groups (beta diversity) were estimated based on weighted UniFrac [39] distances and, sample ordination was visualized using non-metric multidimensional scaling (NMDS). Non-parametric permutational multivariate ANOVA (PERMANOVA) was employed to test the differences in bacterial diversity between groups using *adonis2* [40]. Exploration of taxa was done at the genus level, plotting those that showed less than 0.01 average relative prevalence as ‘Others’. Differential taxa analysis was performed at the species level using linear discriminant analysis (LDA) based on LEfSe [41] in the *microeco* package [42].

## Results

### Feeding increases variability among individuals

**Fig. 1:**
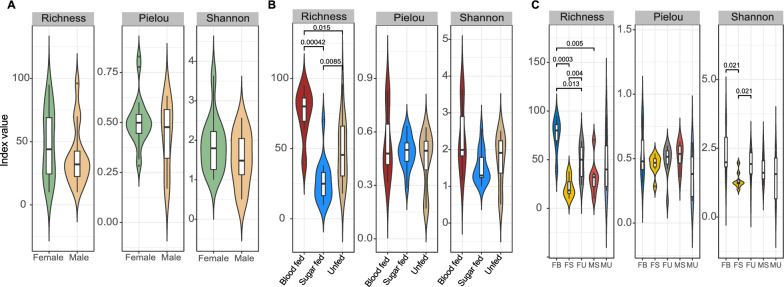
Violin plots for alpha diversity indices observed. Density curves show the distribution of the data among samples of a group. Overlaid box plots summarize the data to show the upper and lower values, and the short horizontal line within each box depicts the mean. Comparison of the index values is between males and females (**A**), feeding statuses (**B**) and sex * feeding groups (**C**)

The variability among sample groups (alpha diversity) was first assessed using estimates of taxa richness, species evenness (Pielou index) and diversity taking into account both evenness and number of species (Shannon–Wiener). Generally, male and female adult mosquitoes showed similar within-group variability (*P* > 0.05) (Fig. 1A). However, when feeding statuses were considered (without separating males and females), the number of bacteria taxa observed was higher among blood-fed than sugar-fed and unfed groups (*P* < 0.01) (Fig. 1B). Despite this, the overall within-group diversity did not differ between feeding groups (Fig. 1B), suggesting that many bacteria were low in abundance bacteria species. Alpha diversity among sexes based on their feeding history was also investigated. Again, blood-fed females showed higher individual-to-individual variability in richness and Shannon diversity than their sugar-fed counterparts (*P* < 0.001) (Fig. 1C), giving first indication of the contribution of blood meals to diversification in microbial composition. While sugar-fed and unfed females showed a difference in richness and within-group diversity, the males were largely the same whether fed or unfed (Fig. 1C).

### Blood-feeding accounts for divergence in the microbiota between male and female mosquitoes

**Fig. 2:**
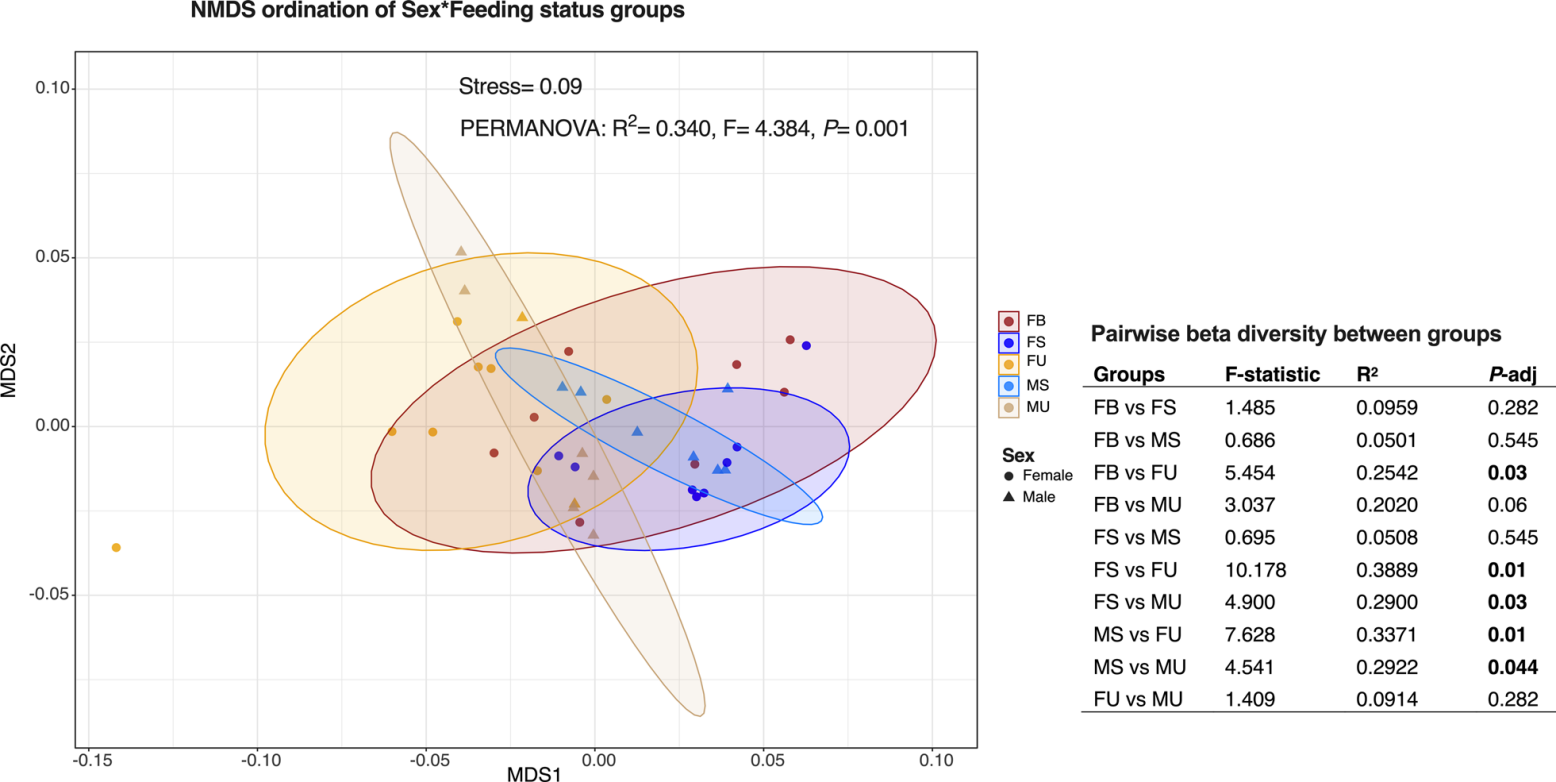
Non-metric multidimensional scaling ordination of samples based on their sex * feeding status. Distances were estimated based on weighted UniFrac and compared with using PERMANOVA. *P*-values are shown in bold where the pairwise comparison is significant with *P*-adjusted value < 0.05. The key to the sample groups are as follows: *FB* female blood-fed, *FS* female sugar-fed, *FU* female unfed, *MS* male sugar-fed, *MU* male unfed

Beta diversity analyses based on weighted UniFrac distances were performed to assess the phylogenetic differences between feeding groups. Overall, male and female adult mosquitoes (considering their feeding statuses) were 34% diverse in their microbial composition (PERMANOVA: R^2^ = 0.340, F = 4.384, *P* = 0.001) (Fig. 2). The source of this diversity was investigated with pairwise comparisons between test groups and, it was observed that the divergence was as low as 5% between sugar-fed females and males (FS vs MS) (*P*-adj = 0.54) and as high as 39% between sugar-fed and unfed females (FS vs FU: *P*-adj = 0.01) (Fig. 2). The difference in diversity between unfed and sugar-fed was significant in both sexes, but higher among females than males (FS vs FU = 39%, MS vs MU = 29%). The ingestion of blood reduced the microbial distance between fed and unfed mosquitoes (FB vs FU) to 25% (*P*-adj = 0.03). Blood-fed females shared phylogenetically similar bacteria with both sugar-fed males and females (Fig. 2).

### *Elizabethkingia meningoseptica* was a major discriminant species for fed mosquitoes

**Fig. 3:**
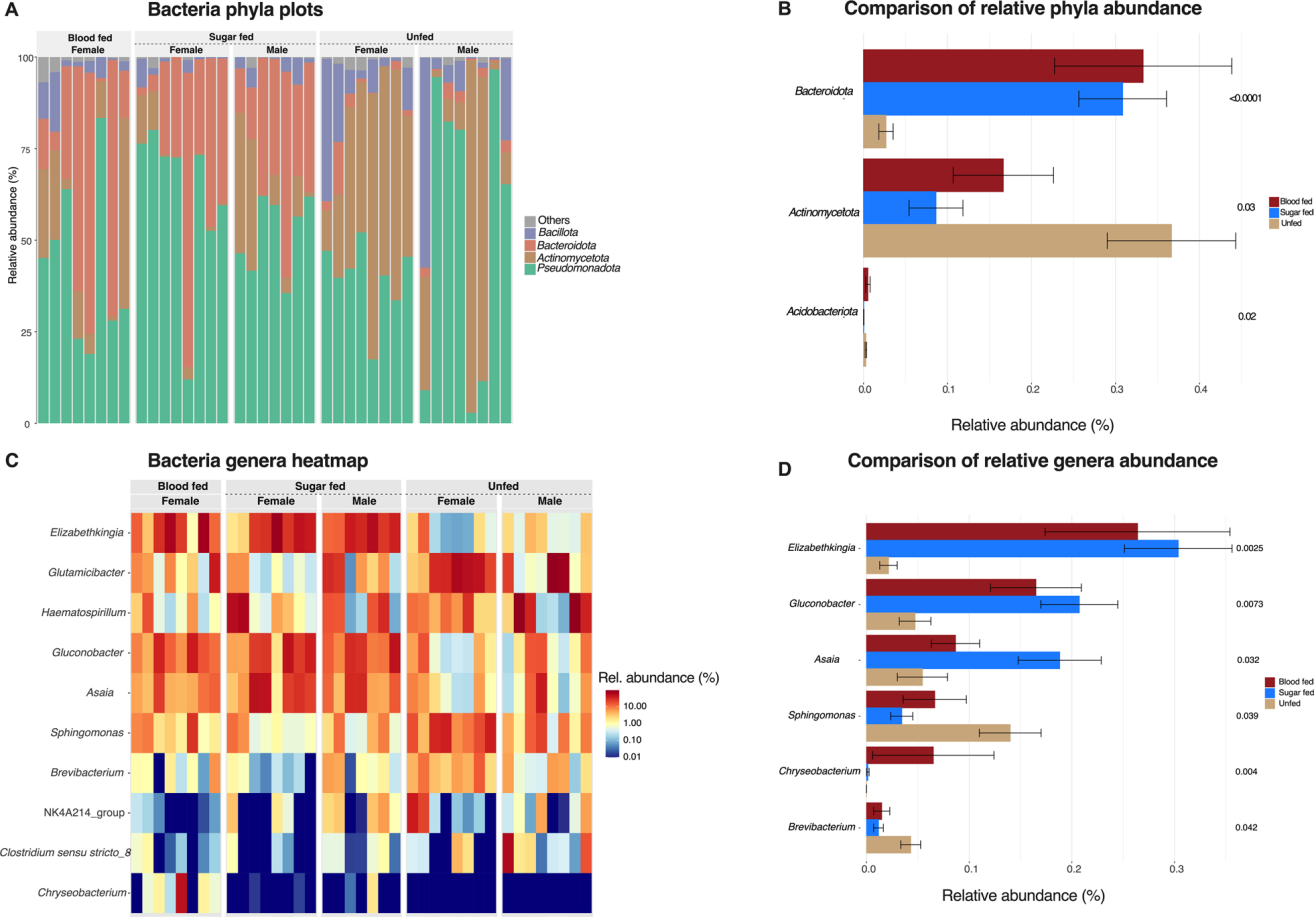
Bacteria phyla and genera abundance. The distribution of taxa with average relative abundance > 1% among test groups is shown as bar plots for phyla (**A**) and heatmap for genera (**C**). Relative abundances are compared between feeding statuses with LEfse (**B**, **D**). The significances shown are adjusted *P*-values

Five phyla accounted for the majority (~ 99%) of the bacteria in the samples examined (Fig. 3A). *Pseudomonadota* (formerly *Proteobacteria*) alone averaged 47% while *Bacteroidota* (formerly *Bacteriodetes*) and *Actinomycetota* (formerly Actinobacteria) both comprised 23% and 21%. The relative abundance of *Bacteroidota* and *Actinomycetota* differed between feeding statuses, the former increasing significantly among fed mosquitoes while the latter increased in newly unfed ones (Fig. 3B). Out of 241 genera identified, only 10 including *Asaia*, and *Elizabethkingia*, were above 1% average relative abundance (Fig. 3C). *Elizabethkingia*, *Gluconobacter* and *Chryseobacterium* were the only highly abundant genera with significantly increased abundance in the fed compared to unfed mosquitoes (Fig. 3D).

**Fig. 4:**
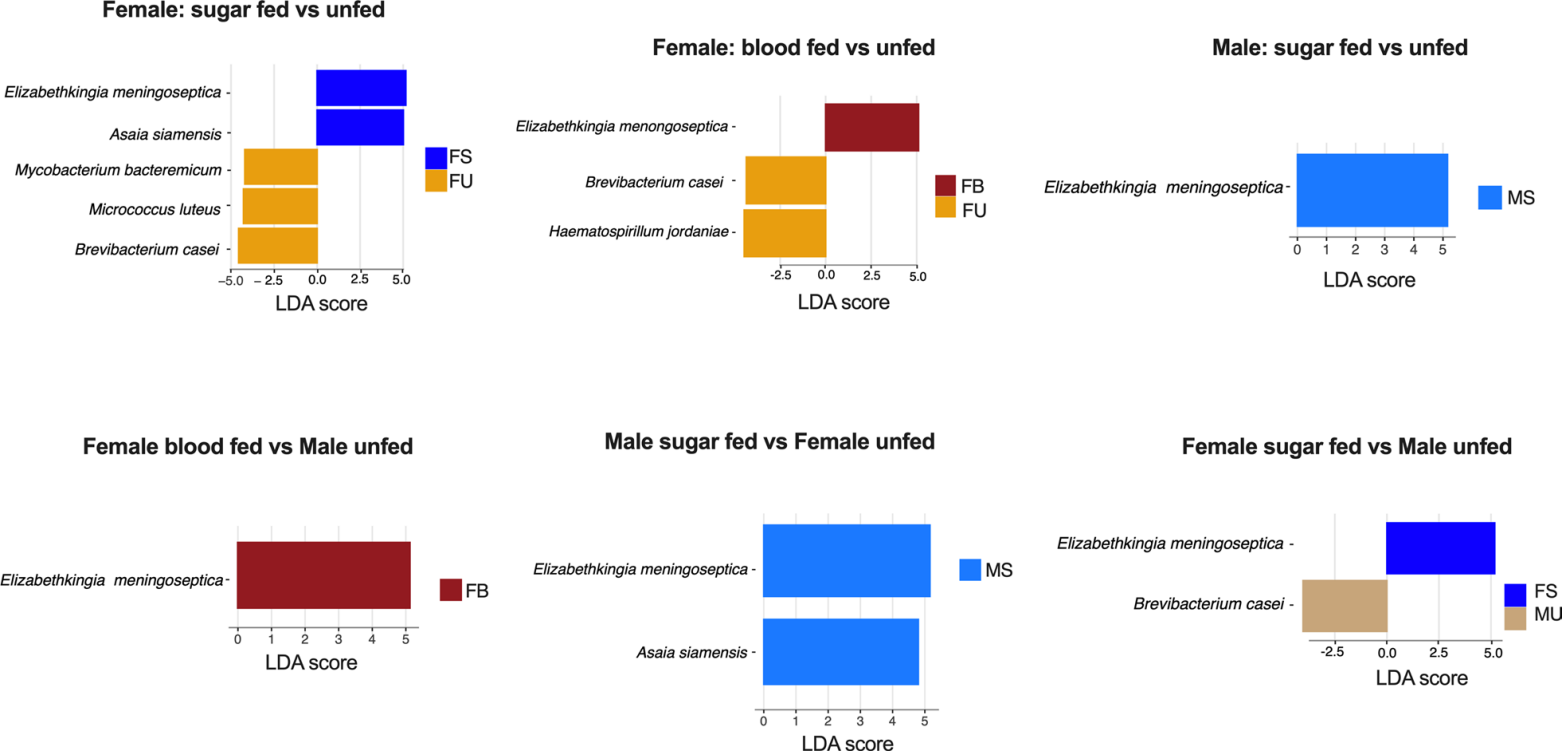
Differential abundance of bacteria species between sex * feeding groups. Plots show only significant species with discriminant scores above 4 (LDA > 4). The key to the sample groups are as follows: *FB* female blood-fed, *FS* female sugar-fed, *FU* female unfed, *MS* male sugar-fed, *MU* male unfed

Based on previous observations that the sex of the mosquito confers different degrees of divergence in the microbiome after feeding (Fig. 2), LefSe [41] analyses were repeated between sex * feeding group pairs to detect bacteria species that drive these differences. To make the analyses more stringent, the discovery of significant taxa was set at an LDA threshold of 4. In all the pairs that showed diverse microbiomes (Fig. 2), *Elizabethkingia meningoseptica* was a significant discriminant bacterium (LDA scores ~ 5) that increased in both sugar-fed and blood-fed mosquitoes (Fig. 4). It was the only bacterium that differentiated unfed from sugar-fed males, and males from females. Whether male or female, sugar-feeding also significantly increased *Asaia siamensis* and was the only species that distinguished the microbiome of sugar-fed from unfed mosquitoes (Fig. 4). *Brevibacterium casei* is shown as a major bacterium in all unfed mosquitoes.

## Discussion

Several bacteria species have been identified, isolated and assessed for their impact on *Anopheles* vector competence [19]. Investigations are often focused on females where the effects of the bacteria on ingested parasites, fertility, fecundity and mortality are evaluated since they have crucial implications on the choice of microbe for disease control strategies. It will also be beneficial to the sustainability of a microbial-mediated strategy if the selected candidate is common to both males and females and propagates readily. These are what make *Microsporidia MB* a good candidate for paratransgenesis and/or for enhancing natural parasite-blocking within mosquito populations [43]. However, before the discovery of *MB*, several bacterial candidates had been identified and investigations are still ongoing into their feasibility for use in disease control. This study demonstrates the feeding-influenced dynamics of two potential bacterial candidates for mosquito biocontrol strategies, *Elizabethkingia* and *Asaia*, in both male and female mosquitoes. In a largely similar microbiome structure of male and female *Anopheles gambiae* mosquitoes, *Elizabethkingia* and *Asaia* are lowly represented in newly emerged adults but increase following feeding. Both species are significant drivers of microbial diversity between adults of unfed and fed statuses. Both males and females had similar magnitude of increase in *E. meningoseptica* after a sugar meal, and in females, the average abundance did not change significantly after blood feeding. *Asaia siamensis*, however, increased substantially higher in sugar-fed females than males but reduced in the former following a blood meal. The implications of these on the use of both bacteria species in disease control are discussed.

The term ‘core microbiome’ is still debatable as it suggests certain bacterial species make up the microbiota of every mosquito species. There are nonetheless some microbes that are commonly identified in almost every mosquito species examined, making them interesting candidates for paratransgenesis mosquito-borne disease control. *Elizabethkingia* is found associated with mosquitoes *Anopheles* [44, 45], *Aedes* [46] and *Culex* [47]. *Elizabethkingia anophelis* and *E. meningoseptica* have been isolated from all life stages of mosquitoes, and from breeding water [48]. When introduced into larvae of *Anopheles stephensi* and *An. gambiae* via ingestion, *E. anophelis* infects the midgut quickly within 3 h with a digestible rate of up to 70% [49]. Similar to what this present study showed, males and females did not differ in their infection density of *Elizabethkingia* and blood feeding increased the cell numbers [49]. This study presented here did not focus on any particular body tissue of the mosquito but the increase in *E. meningoseptica* following sugar and blood feeding suggests their localization in the midgut, which is consistent with studies that observed the dynamics of *Elizabethkingia* in the midguts [48]. *Elizabethkingia meningoseptica* can also inhabit the reproductive organs [50], but it is unknown whether feeding would affect their abundance in these tissues through a systemic mechanism. Although *E. anophelis* and *E. meningoseptica* negatively affect the development of *Plasmodium* [45, 51], they are virulent when injected into the haemolymph [50] suggesting that their use as biocontrol agents would have to be carefully formulated such that they remain symbiotic.

*Asaia* is also found in many mosquito species across zoogeographical locations in many mosquito species and inhabits the salivary glands, guts and reproductive tissues of both males and females [24, 52, 53]. They can be horizontally transferred into the guts via nectar sources [27] and, when located in the reproductive tissues are transmissible from male to female adult during mating and to offspring from the female parent [24, 54]. Their vertical and horizontal mode of transmission will ensure their self-propagation when modified and introduced into populations. For example, when introduced through larval feeding *Asaia* sp. transstadially inhabited the midguts of adults and were passed to the next generation [55]. In the current study, *Asaia simensis* was a discriminant species between unfed and sugar-fed females, but not between males. It may suggest that the tissue localization of *Asaia* differs between male and female *Anopheles* mosquitoes with a relatively high abundance in male reproductive tissues while the female has its midgut more highly populated. The implications of this for the use of *Asaia* spp in control interventions is that sugar feeding would lead to their proliferation in females but not males. The *An. gambiae* mosquito can, however, be inhabited by multiple strains of *Asaia* sp. [52] which are localized in different tissues [56]. It is unknown, though, whether different strains exhibit tissue tropism and are host-sex specific as that would suggest that multiple *Asaia* species would be required for biocontrol methods. This merits further investigations to determine whether there could be differences in how male and female mosquitoes acquire and use *Asaia*.

## Conclusion

Two candidates for bacteria-mediated parasite transmission blocking in *An. gambiae* mosquitoes, *Elizabethkingia* and *Asaia*, are stable in adult mosquitoes through sugar and blood feeding. Further studies are recommended for a clearer understanding of their multiple species infections and tissue tropism in the male and female hosts, to inform the most appropriate species to use, the best approach to introduce them into the mosquito population and their propagation mechanisms. It will also be very useful to assess life table parameters associated with the presence of these bacteria as major symbionts in the mosquito host.

### Supplementary Information

**Additional file 1.** Feeding status and sex of mosquito samples used in the study.

## Data Availability

All data generated or analysed during this study are included in this published article and its Additional files. The demultiplexed sequence reads generated in this study are available in the NCBI SRA database under BioProject PRJNA1045275.
